# Ranolazine in the prevention and treatment of atrial fibrillation: A protocol for meta-analysis

**DOI:** 10.1097/MD.0000000000025437

**Published:** 2021-04-23

**Authors:** Chengdai Yuan, Wei Luo, Xiaocao Ren, Maxiao Ya, Wenlong Yan, Quanbin Hui

**Affiliations:** a Department of Pharmacy, Baoji Traditional Chinese Medicine Hospital, Baoji, Shanxi; b The Second School of Clinical Medicine; c The First School of Clinical Medicine, Lanzhou University; d Baoji People's Hospital, Baoji, Shanxi, China.

**Keywords:** atrial fibrillation, meta-analysis, ranolazine

## Abstract

**Background:**

Atrial fibrillation (AF) is the most common clinical arrhythmia and a major cause of morbidity and mortality in clinical practice. This study aims to determine the ranolazine for prevention and treatment of atrial fibrillation.

**Method:**

This study adheres to the Preferred Reporting Items for Systematic Reviews and Meta-analysis for Protocols. Chinese electronic Database (CBM, Wanfang, and CNKI) and international electronic databases (PubMed, Embase, Cochrane Library, and Web of Science) will be searched for all relevant published articles. We will apply no language or the year of publication restrictions. Study selection, data collection, and assessment of study bias will be conducted independently by a pair of independent reviewers. The Cochrane risk of bias (ROB) tool will be used for the risk of bias assessment. The quality of evidence will be evaluated by Grading of Recommendations Assessment Development and Evaluation (GRADE) system. The statistical analysis of this meta-analysis will be calculated by Review manager version 5.3.

**Results:**

The results of this study will be published in a peer-reviewed journal.

**Conclusion:**

This review will evaluate the value of ranolazine interventions for patients with AF, and provide meaningful conclusions or high-level evidence for clinical practice and further research.

**Trial registration:**

This study protocol was registered in open Science framework (OSF), (Registration DOI: 10.17605/OSF.IO/T6W9Q).

## 1 Introduction

Atrial fibrillation (AF) is the most common persistent arrhythmia, which is characterized by activation of atrial disorders, leading to loss of atrial mechanical function and irregular ventricular response.^[1]^ AF is most common in adults and its incidence increases significantly with age,^[2]^ related to morbidity, mortality, and high health care costs. Studies have shown that the lifetime risk of atrial fibrillation in people aged 40 or older is about 1/4.^[3]^ As the population aging, the incidence of AF is increasing^[4]^ and the prevalence rate of males is slightly higher than that of females. AF can lead to serious complications, such as heart failure, stroke, and other thromboembolic events.^[5]^ Compared with the general population, the risk of stroke increased fivefold, the risk of heart failure tripled, and the risk of death tripled.^[6]^ There are 5 different types of AF, including first-diagnosed AF, paroxysmal AF, persistent AF, long-standing persistent AF, and permanent AF.^[5]^ In AF patients, reduced atrial contraction and irregular pulse lead to reduced cardiac output, resulting in decreased physical strength and quality of life (QoL),^[7]^ some patients have palpitation, shortness of breath, fatigue, dizziness and fainting (fainting).

At present, the treatment of AF is mainly focused on reestablishing and maintaining the rhythm of atrial fibrillation (rhythm control) and protecting patients from thromboembolic complications and reducing symptoms and discomfort associated with AF.^[8]^ Catheter ablation and antiarrhythmic drugs are the most common treatments, but drugs are not always effective and have high rates of side effects if long term use. A meta-analysis of nonrandomized and randomized studies of all antiar-rhythmic drugs showed an average success rate for prevention of atrial fibrillation recurrence of 52% over 1 year.^[9]^ Consequently, there is a need for more effective and safe methods for patients with AF to manage their condition.

Ranolazine, an inhibitor of late inward sodium current, is approved in the united States and Europe as a second-line agent for use in people with stable angina.^[10]^ Although several pharmacological activities of ranolazine have been described in some study, the precise way the drug works for AF patients is not fully understood. And several studies showing some inconsistencies in terms of its magnitude and direction of the effect.^[11,12]^ Considering management of patients with AF can reduce their risk of death, hospitalization, and health care costs, and there is no consensus on the optimal treatment options for the ranolazine in patients with AF, we will perform a comprehensive meta-analysis to evaluate the efficacy of ranolazine in this population.

## 2 Materials and methods

### 2.1 Study registration

This protocol will be reported according to preferred reporting items for systematic review and meta-analysis protocols (PRISMA-P).^[13]^ As a part of our study, this study protocol has been registered on the open Science framework (OSF) (Registration DOI: 10.17605/OSF.IO/T6W9Q).

### 2.2 Search strategy

In this study, we will search 4 international electronic databases (PubMed, Embase, Cochrane Library, and Web of Science) and 3 Chinese electronic databases (Chinese Biomedical Databases (CBM), Wanfang database, and China National Knowledge Infrastructure (CNKI)). Our search did not restrict publication type, or year of publishing. The search terms and basic search strategy were as follows: (Ranolazine OR ranexa OR GB∗ OR astrocyt∗ OR GBM∗) AND (Atrial Fibrillation OR Atrial Fibrillation ∗ OR atrium fibrillation∗ or atrial ablation∗ or atrial next arrhythmi∗) AND (ranolazine OR ranexa OR 110445-25-5 OR latixa) AND random∗. In addition, to ensure a comprehensive data collection, references of relevant reviews, grey literature will be searched manually to identify additional eligible studies. We will provide specific search strategy of PubMed and will be shown in Appendix 1.

### 2.3 Selection criteria

#### 2.3.1 Types of studies

Randomized controlled trials (RCTs) comparing ranolazine with antiarrhythmic drugs or active intervention in patients with AF will be included. We excluded nonrandomized studies (for example, studies with evidence of inadequate sequence generation such as alternate days, patient numbers) and cluster-RCTs.

#### 2.3.2 Types of participants

We will include adults (aged 30 years or older) with AF and the diagnosis criteria according to the AHA/ACC/HRS 2019 Atrial Fibrillation Treatment Guidelines.^[14]^ we will exclude younger participants because of the extremely low incidence of AF in this population.

#### 2.3.3 Types of interventions

Any RCTs involving ranolazine as treatment for AF. Interventions will be included but were not limited to the following:

1. ranolazine vs placebo for AF;
2. ranolazine vs no treatment for AF;
3. ranolazine vs antiarrhythmic drugs or any other conventional treatment.

#### 2.3.4 Types of outcome measures

We will plan to assess any studies including at least one of the following outcomes. The primary outcome includes the

1. clinical efficacy,
2. all-cause mortality,
3. adverse effects (refer to withdrawals from taking the study drug caused by adverse events),
4. stroke and thromboembolic events.

The secondary outcomes consist of

1. Heart failure,
2. Recurrence of atrial fibrillation,
3. Health-related quality of life (HRQoL) (measured with a validated quality of life questionnaire such as EQ-5D or Short Form-36 (SF-36),
4. economic costs of the intervention (cost-effectiveness),
5. patient satisfaction.

### 2.4 Exclusion criteria

The following criteria will be excluded:

1. the study included too little information or data could not be obtained, such as review articles, editorials, comments, and protocols;
2. conference abstracts, and duplicate reports of the same study.

### 2.5 Study selection

Two reviewers will independently review the title and abstracts of initially selected studies according the inclusion and exclusion criteria. The full texts of articles will be retrieved if there was any doubt about inclusion of the study. If necessary, any disagreements regarding inclusion will resolved through discussion or by consulting a third reviewer. We will record the selection process in sufficient detail to complete a PRISMA flow chart.

### 2.6 Data extraction

After selecting studies based on the inclusion and exclusion criteria, 2 authors (YCD and LW) will use the prepiloted standardized data extraction form to independently extract data from included studies. We will resolve any disagreements by discussion and consultation with a third review author (LWH). The specific characteristics extracted will included the first author, year of publication, study country, the participant numbers in the ranolazine group and control group, details of the ranolazine intervention and control group, outcome measures, and adverse events reported. If the information present was unclear or if information was missing, the corresponding author of the study was contacted via email.

### 2.7 Assessment of risk of bias in included studies

Two review authors (RXC and MXY) will use the Cochrane risk of bias tool to independently assess the risks of bias of each included study.^[15]^ We will assess the risk of bias in 7 domains: sequence generation, allocation concealment (selection bias), blinding of participants and personnel (performance bias), blinding of outcome assessors (detection bias), incomplete outcome data (attrition bias), selective outcome reporting, and other potential sources of bias. We will judge each item as being at yes (“low risk of bias”), no (“high risk of bias”), or unclear (“moderate risk of bias”). We will resolve any disagreement in bias classification by discussion to reach consensus and, if necessary, by discussion with a third review author.

### 2.8 Data synthesis and analysis

We will use Review Manager 2020 to synthesize the available data. We will calculate dichotomous outcomes as risk ratios (RRs) with 95% confidence intervals (CIs). For continuous outcomes (e.g., QoL scores), we will calculate mean difference (MD), or standardized mean difference (SMD) for the same continuous outcome measured with different metrics, and the 95% CIs. For time-to-event outcomes (e.g., overall survival), we will calculate the hazard ratio (HR) with its 95% CIs. If we are unable to perform a meta-analysis due to substantial differences between included studies, we will perform a narrative synthesis of the data.

### 2.9 Subgroup analysis and investigations of heterogeneity

We will investigate the potential sources of heterogeneity in the results for each method using subgroup analyses or meta-regression, depending on the number of studies identified and the nature of the source of heterogeneity. We will assess the clinical heterogeneity of the included studies by comparing participants’ characteristics (age, gender, and type of AF), interventions (administration method, dosage, frequency and duration, control intervention). We will assess statistical heterogeneity among the included studies using the Chi^2^ test and the *I*^2^ statistic. When the *I*^2^ statistic value is greater than 50% (substantial heterogeneity), we will perform subgroup and sensitivity analyses to consider possible reasons for heterogeneity.

### 2.10 Sensitivity analyses

We plan to perform a sensitivity analysis restricting the analysis to studies we judge to be at low or unclear risk of bias.

### 2.11 Certainty assessment

Two trained GRADE methodologists will use Grades of Recommendation, Assessment, Development, and Evaluation (GRADE) system^[16]^ to assess the certainty (quality) of evidence associated with specific outcomes and constructed a summary of findings table. Assessment of the quality of evidence considers study methodological quality, directness of the evidence, heterogeneity of data, precision of effect estimates, and risk of publication bias.^[17]^ For each significant outcome, we will be awarded 4 points to begin with as these were based on randomized trials and assessed the limitations that can reduce the quality of this evidence.

### 2.12 Ethics and dissemination

This study belongs to the category of systematic review and it is only a secondary analysis of the published data, so ethical approval is not applicable to this study.

## 3 Discussion

Atrial fibrillation is currently the most common serious arrhythmia, with a prevalence of 1% to 2% in the general population, and the incidence increasing with age. Therefore, the purpose of this review was to explore the benefits and harms of ranolazine in people with AF, summarize current evidence for the incremental resource use, utilities, costs and cost-effectiveness associated with this drug, and provide meaningful conclusions for clinical practice and further research.

## 4 Strengths and limitations of this study

This will be the latest systematic review investigating ranolazine for the treatment of people with AF. At present, there is no clear consensus on how to use existing evidence to guide individual treatments seen in the clinic. This study will provide insights for planning future studies. Moreover, compared with previous systematic reviews, this study takes into account a wider range of outcome indicators (clinical efficacy, all-cause mortality, adverse effects, stroke and thromboembolic events, heart failure, recurrence of atrial fibrillation, economic costs of the intervention, etc). The limitations of this study include: the number of studies that meet our screening criteria may be limited; due to language barriers, only the 2 languages of the trial are included, and other related studies may be missing.

## Author contributions

**Conceptualization:** Chengdai Yuan, Xiaocao Ren, Weihai Liu.

**Data curation:** Wei Luo, Wenlong Yan.

**Formal analysis:** Wei Luo.

**Funding acquisition:** Weihai Liu.

**Investigation:** Xiaocao Ren, xiaoya Ma.

**Methodology:** Xiaocao Ren, Wenlong Yan.

**Project administration:** Weihai Liu.

**Resources:** Xiaocao Ren, xiaoya Ma, Wenlong Yan.

**Software:** Xiaocao Ren, Wei Luo, xiaoya Ma, Wenlong Yan.

**Supervision:** Chengdai Yuan.

**Validation:** Wenlong Yan.

**Visualization:** Chengdai Yuan.

**Writing – original draft:** Chengdai Yuan.

**Writing – review & editing:** Chengdai Yuan, Weihai Liu.

## Supplementary Material

Supplemental Digital Content
