# Supplementary material for: Ranolazine in the prevention and treatment of atrial fibrillation: A protocol for meta-analysis
Source: Medicine (Baltimore). 2021 Apr 23;100(16):e25437. doi: 10.1097/MD.0000000000025437 (PMC8078434; doi:10.1097/MD.0000000000025437)
Supplement: Supplemental Digital Content [file medi-100-e25437-s001.docx]

**The specific search strategy will be (taking PubMed as an example):**

1. Atrial Fibrillation [mh]

2. (Atrial Fibrillation* OR atrium fibrillation* or atrial ablation* or atrial next arrhythmi*)). ti,ab.

3. 1 or 2

4. (Ranolazine OR ranexa OR GB* OR astrocyt* OR GBM*). ti,ab.

5. 3 and 4

6. randomized controlled trial[mh]

7. randomized controlled trial.pt.

8. controlled clinical trial.pt.

9. randomized.ab.

10. placebo.ab.

11. clinical trials as topic.sh.

12. randomly.ab.

13. trial.ti

14. 6 or 7 or 8 or 9 or 10 or 11 or 12 or 13

15. (animals not (humans and animals)).sh.

16. 14 not 15

17. 5 and 16
